# Sustained Effectiveness of Evidence-Based Parenting Programs After the Research Trial Ends

**DOI:** 10.3389/fpsyg.2018.02035

**Published:** 2018-10-26

**Authors:** Gemma R. Gray, Vasiliki Totsika, Geoff Lindsay

**Affiliations:** ^1^Centre for Educational Development, Appraisal and Research (CEDAR), University of Warwick, Coventry, United Kingdom; ^2^Department of Psychology, University of Warwick, Coventry, United Kingdom; ^3^Centre for Developmental Psychiatry and Psychology, Monash University, Melbourne, VIC, Australia

**Keywords:** parenting programs, effectiveness, child behavior, sustained implementation, evidence-based practice

## Abstract

Despite ample evidence of the efficacy and effectiveness of evidence-based parenting programs (EBPPs) within research-led environments, there is very little evidence of maintenance of effectiveness when programs are delivered as part of regular service provision. The present study examined the effectiveness of EBPPs provided during a period of sustained service-led implementation in comparison to research-led effectiveness evaluation. Data from 3706 parents who received EBPPs during sustained implementation by services were compared to data from 1390 parents who had participated in an earlier researcher-led effectiveness trial of a national roll-out of EBPPs in England. In both phases, parents completed measures of child behavior problems, parenting style and parental mental well-being prior to starting parenting programs (pre-test), at the end of the programs (post) and at 12-months follow up. Results from Generalized Estimating Equations controlling for potential covariates indicated significant improvements in child behavior problems during sustained implementation, similar to the effectiveness phase; significant improvements in parenting style which were larger than the effectiveness phase at 12-month follow up; and significant improvements in parental mental well- being. Our findings demonstrate effective maintenance of gains when EBPPs are provided as part of regular provision across a large sample of English parents. Successful long-term implementation should consider effectiveness of EBPPs across the population, given the large contextual changes that take place between researcher-led evaluations and service take-up. Our findings support the integration of EBPPs in public health approaches to addressing child behavior problems and parent well-being.

## Introduction

Evidence-informed policy making in public health or specialist service provision relies, in part, on research evidence about the efficacy and effectiveness of available interventions (Davies et al., 2000). According to recent standards of evidence (Gottfredson et al., 2015), interventions are gradually developed by building up the evidence in relation to the core mechanism of change, testing the efficacy of the full intervention package in controlled conditions, and then examining the effectiveness of the intervention under conditions that resemble real-life conditions more closely (Flay et al., 2005; Gottfredson et al., 2015).

Within implementation science it has become recognized that this evidence pathway should not end after demonstration of efficacy and then effectiveness when the intervention is scaled up and implemented as part of regular service provision. Rather, models of evidence pathways have extended to a further stage of sustainment or sustainability (Aarons et al., 2011). However, the theoretical conceptualization of this stage has been contentious, as indicated by the use of both sustainment (a state) and sustainability (a characteristic of the implementation). For example, in their review of 125 studies Wiltsey Stirman et al. (2012) found that 62% of studies used the term “sustainability”; that only 36 (29%) defined sustainability; and that there were a number of different definitions, the most common of which was that by Scheirer (2005) which just eight studies used. A recent review demonstrates consistency has not improved (Moore et al., 2017).

The main focus of such studies has been on the influences that enhance or reduce sustainability. A systematic review by Durlak and DuPre (2008) identified 23 contextual factors that can influence the success of an implementation. Wiltsey Stirman et al. (2012) identified four main categories with 24 subsidiary categories: innovation characteristics, e.g., fit, ability to maintain fidelity/integrity; context, e.g., climate, leadership, setting characteristics and system/policy change; capacity, e.g., funding, workforce (staffing attributes and community/stakeholder support); and processes and interactions, e.g., training and education, ongoing support, and engagement/relationship building.

In both these reviews, intervention effectiveness was not among these factors. Demonstration of effectiveness during sustained implementation was considered relevant only to service providers as a means of monitoring performance and assuring quality (Franks and Schroeder, 2013). In addition only 22% of the studies reviewed by Wiltsey Stirman et al. (2012) reported sustainability of individual outcomes. Shediac-Rizkallah and Bone (1998) noted inconsistencies with the definition of sustainability, and distinguished between six definitions of sustainability. Here we focus on definition three:

*“A development program is sustainable when it is able to deliver an appropriate level of benefits for an extended period of time after major financial, managerial and technical assistance from an external donor is terminated (United States Agency for International Development, 1988)*” (Shediac-Rizkallah and Bone, 1998, pp. 91).

Indeed, findings from small-scale studies that examined effectiveness during transition to service-led provision (Zubrick et al., 2005; Price et al., 2012; Skar et al., 2015) highlighted the importance of maintaining effectiveness during sustained implementation.

The need to monitor the effectiveness of parenting programs during sustained implementation extends beyond the needs of a particular service provider: it should concern evidence-informed policy-making. Moreover, program delivery changes as the level of experimental control changes, as well as many factors associated with successful implementation (c.f., Durlak and DuPre, 2008), so a change in the level of effectiveness should be considered a likely characteristic of sustained implementation, along with recognition of the limitations of our systemic capacity to control all factors we know are related to successful implementation. Therefore, to support a public health approach to the promotion of EBPPs, the question is no longer whether they work, but whether they still work when they are provided as part of regular service provision across the population.

Therefore the focus of the present study was on the sustainability of effectiveness, defined as maintenance of positive effects of the program(s) at a comparable level to that shown in the earlier formal effectiveness trial; this definition is consistent with Shediac-Rizkallah and Bone (1998).

In the present study we examine the sustainability of the effectiveness of parenting programs once these are implemented as part of regular service delivery in communities, outside trials which had previously demonstrated their effectiveness. Sustainability of effectiveness, therefore, is defined as maintenance of positive effects of the program(s) at a comparable level to that shown in the earlier formal effectiveness trial. The implementation domain is that of parenting programs which aim to reduce behavior problems among children because such problems can persist into adulthood and have both negative consequences for the individuals, and high societal costs (Scott et al., 2001). Parenting programs are mainly based on behavioral science and social learning models (Sanders et al., 2012) and aim to develop more adaptive parenting techniques to help parents to manage their child’s behavior. Numerous randomized controlled trials (RCTs) have established the evidence-base of programs such as Triple P (Sanders et al., 2000) and Incredible Years (Webster-Stratton et al., 2001). Systematic reviews and meta-analyses of these RCTs have concluded that parenting programs are effective interventions to reduce child behavior problems and improve the overall emotional and behavioral adjustment of children, increase positive parenting styles, decrease ineffective use of discipline, and improve maternal mental health (Kaminski et al., 2008; Nowak and Heinrichs, 2008; Dretzke et al., 2009; Barlow et al., 2012, 2016; Furlong and McGilloway, 2015).

Whereas this evidence-base is key for policy makers wishing to embed parenting programs into regular service provision, it is not sufficient. The next step is to demonstrate that parenting programs work equally well when implemented under real-world conditions, gradually building from smaller effectiveness demonstrations to larger-population effectiveness research (scaling up). In the United Kingdom, an example of the latter is the United Kingdom-government instigated and funded Parenting Early Intervention Pathfinder evaluation (2006–2008) of three evidence-based parenting programs (EBPPs) across 18 Local Authorities (LAs; geographical regions with administrative powers) in England. On the basis of evidence of their effectiveness (Lindsay et al., 2011b) the funder rolled out eight EBPPs across all 152 LAs in England, the Parenting Early Intervention Programme, and evaluated their effectiveness in a sample of 43 LAs (Lindsay et al., 2011a; Lindsay and Strand, 2013). The Lindsay et al. (2011a) study demonstrated that rolling out EBPPs was associated with small to moderate reductions in child behavior problems, and large changes in parenting style and parental mental well-being. In the United States, the rolling out of Triple P across 18 counties in South Carolina further demonstrated the public health benefits associated with the prevention of child maltreatment (Prinz et al., 2009).

Although there is limited evidence on the sustainability of EBPPs with respect to individual outcomes, findings from small-scale studies that examined effectiveness during transition to service-led provision have provided indicative evidence of positive effects. For instance, Price et al. (2012) focused on externalizing behaviors of children in foster care and found that provision of the parenting intervention by community agencies was as effective at reducing the target behaviors as an earlier effectiveness trial. Skar et al. (2015) assessed the long term sustainability of effects in a community-wide parenting program, finding that the beneficial effects on parenting measures were maintained 6 and 12 months later.

Hence there is a general need to monitor the effectiveness during sustained implementation which extends beyond the requirements of a particular service provider: it should concern evidence-informed policy-making that is looking to maintain service provision and well-being levels of the population. A change in effectiveness during implementation may be considered likely, given the large number of factors that may influence sustainability of effectiveness (Durlak and DuPre, 2008). Therefore, to support a public health approach to the promotion of EBPPs, the question concerning effectiveness applies not only to whether they are effective in trials, but also to whether they remain effective when provided as part of regular service provision across the targeted population.

The aim of the present study was to examine the effectiveness of EBPPs during a period of sustained implementation by services in England. The study responds to recent calls by the Society for Prevention Research (Gottfredson et al., 2015) to develop robust evidence of effectiveness during scaling-up of interventions. Our main research question examined whether effectiveness of EBPPs could be maintained during the phase of service-led sustained implementation as compared to an earlier researcher-led effectiveness evaluation phase. Specifically, we examined whether changes in child behavior problems, parenting style and parental mental well-being were significantly different between service-led sustained implementation and the previous researcher-led effectiveness evaluation.

## Materials and Methods

### Design

**FIGURE 1:**
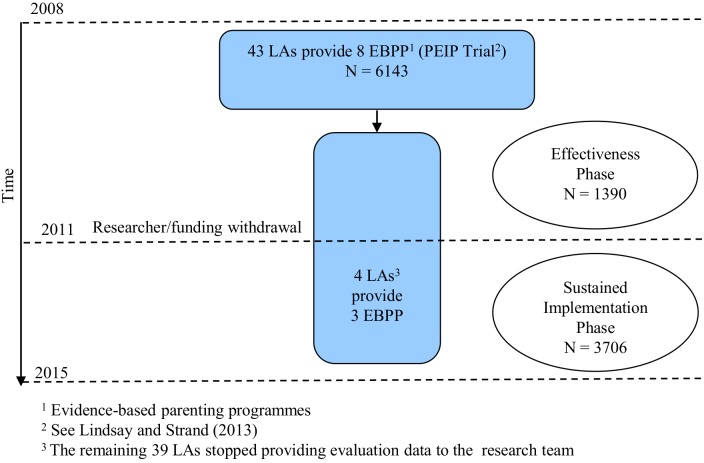
Study design.

This study compares the effectiveness of parenting programs delivered across two different phases: the effectiveness trial comprised a researcher-led evaluation of effectiveness during national roll-out of parenting programs across England (2008–2011; Lindsay and Strand, 2013); following this, the service-led sustained implementation phase (2011–2016) included service evaluation data collected for service monitoring purposes (see Figure 1). In the present study, data were drawn from four LAs that participated across both phases. These LAs requested that the research team continued to collect data from parents enrolling during the sustained implementation phase. Apart from providing annual reports of the analysis of their results, we took no part in the LAs implementation of the EBPPs that they had selected for delivery. The sustained implementation phase started in the school year following the last year of the trial implementation phase.

### Participants

Parents were recruited to parenting programs through multiple routes, which were comparable in each LA. The recruitment was based entirely on the parent’s or professional’s concern about the child(ren)’s behavior. Local authorities were quite liberal with their inclusion criteria, including those parents who self-referred, and referrals from schools, social services, and health services, with the agreement of the parent. No LA had formal inclusion or exclusion criteria. There were no inclusion or exclusion criteria for the research: all parents for whom pre-course data were available were included in the trial. These criteria applied to both phases of the study. Table 1 presents the demographic information for the 1390 parents who took part in the effectiveness trial phase, and 3706 from the sustained implementation phase.

**Table 1:** Characteristics of parenting program participants during the effectiveness phase and the sustained implementation phase.

Variable	Effectiveness trial phase (*N* = 1390)	Implementation phase (*N* = 3706)	Comparison
Parent female gender (%)	88.29	82.73	*x^2^* = 21.33, *p* < 0.001
Parent has no educational qualifications (%)	19.11	16.35	*x^2^* = 1.90, *p* = 0.092
Median parent support need (SD)^1^	1 (1.02)	1 (1.06)	*U* = 2420537, *p* < 0.001
Parent is White British (%)	51.20	70.64	*x^2^* = 187.01, *p* < 0.001
Family rents the home (%)	66.15	65.62	*x^2^* = 2.83, *p* = 0.050
Free school meal eligibility (%)	50.65	44.14	*x^2^* = 17.21, *p* < 0.001
Single parent status (%)	38.81	35.75	*x^2^* = 1.04, *p* = 0.162
Median socio-economic deprivation (SD)^2^	2 (0.87)	2 (0.81)	*U* = 2489623, *p* = 0.059
Child male (%)	60.97	56.37	*x^2^* = 0.21, *p* = 0.335
Child mean age (SD)	7.08 (4.19)	6.94 (4.27)	*F*(1,4628) = 0.92, *p* = 0.337
Child has special educational needs (%)	10.43	11.95	*x^2^* = 2.29, *p* = 0.074
**Program**			
Incredible years (%)	3.38	0.27	
Triple P (%)	89.28	92.88	
STOP (%)	7.34	6.85	*x^2^* = 89.23, *p* < 0.001


*^*1*^Parent support need reflects the number of health/social care professional’s parents received support from over the past 6 months (range 0–5).*

*^*2*^Socio-economic deprivation is a composite of parent education, housing, family structure and free school meal status. Higher scores indicated higher deprivation.*

### Measures

#### Demographic Information

Demographic information was collected prior to the start of parenting programs and included child age, gender and Special Educational Need (SEN) status, parent gender, parents’ contact with health and social care professionals, parent education, ethnicity, family structure, housing status, and whether their child was eligible for free school meals. SEN status indicates that the child has been assessed by the educational authority as not being able to learn in the same way or at the same pace as his peers, and that additional or differentiated provision is needed to address the child’s learning needs. Eligibility for free school meals is determined by the family’s income and is a proxy measure of income poverty.

##### Socio-economic deprivation

A composite measure of socio-economic deprivation was developed by combining data on parental education level (no educational qualifications vs. Level 3+), single parent status (single vs. dual parent), home ownership (rent home vs. own home) and free school meal eligibility (eligible vs. ineligible). No educational qualifications indicates exiting school without any General Certificate for Secondary Education (GCSE) level qualifications, i.e., Level 3/upper secondary level, International Standard Classification of Education (ISCED; UNESCO, 2011). Socio-economic deprivation scores ranged from 0 to 4, with higher values indicating more deprivation.

##### Parent support needs

Parents were asked to indicate whether they had contacted professional support for themselves over the past 6 months: medical practitioner, psychiatrist, counselor, social workers or other healthcare and support professionals. We summed the number of contacts indicated to capture the level of support need of the parent. A score of 0 indicated the participant had no contact with any of these professionals and therefore a low ‘support need,’ whereas a score of 5 indicated parents had had contact with all the support professionals, therefore demonstrating a high level of support need.

#### Child Behavior Problems

The Strengths and Difficulties Questionnaire (SDQ; Goodman, 1997) is a well-established measure of children’s behavioral and emotional problems. The SDQ contains 25 items, grouped into five scales (5 items per scale): conduct problems, emotional symptoms, hyperactivity, peer problems, and prosocial behavior. For each item, parents rate their child’s behavior on a 3-point scale (0 = not true, 1 = somewhat true, 2 = certainly true). The first four scales are summed to give a total difficulties score. The SDQ is used extensively in research and clinical practice and has well-established psychometric properties (Goodman, 2001).

We used the 4–17 year-old version of the SDQ, though the SDQ has been validated for use with children aged 2–17 years old ^1^ with altered wording of three of the items for the 2–3 years group. Here we present internal consistency data from the 4–17 years group, though findings were similar for the 2–17 year group. Good levels of internal consistency were demonstrated across both phases for conduct problems (Cronbach’s alphas were 0.71 and 0.69 for the effectiveness and sustained implementation phase, respectively); emotional symptoms (α = 0.69 and 0.73); hyperactivity (α = 0.73 and 0.76) and total difficulties score (α = 0.82 and 0.84). Peer problems and prosocial behavior subscales were not included in the present study as these are behaviors not directly targeted by parenting programs.

#### Parenting

The Parenting Scale – Adolescent (Irvine et al., 1999) is a 13-item scale, shortened from an original 30-item scale (Arnold et al., 1993) to assess parenting style. The original 30-item version of this questionnaire has been validated for use with parents of children aged 18 months to 16 years (Arnold et al., 1993; Karazsia et al., 2008) and reliability and validity of the shorter version have been established for children aged 2 – 16 (Karazsia et al., 2008). Two subscales are available: laxness (six items) and over-reactivity (six items) as well as a single ‘parental monitoring’ item. Parents rate each item on a 7-point scale and the scores in each subscale are summed. Internal consistency was good (laxness, α = 0.75 and 0.83 and over-reactivity, α = 0.72 and 0.81, for each phase, respectively).

#### Parent Mental Well-Being

The Warwick-Edinburgh Mental Well-Being Scale (WEMWBS; Tennant et al., 2007) is a 14-item measure of subjective mental well-being in adults. Parents rated 14 statements on a 1 (none of the time) to 5 (all of the time) scale. Total mental well-being score ranges from 14 to 70, with higher scores indicating higher mental well-being. Internal consistency levels were high: α = 0.93 for the effectiveness trial phase, and α = 0.94 for the sustained implementation phase.

### Parenting Programs

During the effectiveness trial phase, the United Kingdom Department for Education (DfE) selected eight EBPPs, which had been accredited by the National Academy for Parenting Practitioners (NAPP; Asmussen et al., 2012) to a standard of effectiveness determined by the DfE. LAs could provide one or more of these EBPPs. The DfE funded LAs through the Parenting Early Intervention Programme to develop their infrastructure, including strategic and operational lead officers and staff who had been trained to be facilitators of the relevant programs the LAs chose to implement. Training was provided by the program providers (see Lindsay et al., 2011b). During the sustained implementation phase, the LAs chose to continue with the programs. Three parenting programs were common across the two phases: Incredible Years, Triple P and STOP.

#### Incredible Years

The Incredible Years program (IY; Webster-Stratton et al., 2004) was developed for parents of children between 8 and 13 years old and focuses on teaching parents how to manage the child’s behavior problems through improved parenting. In the current study, providers of IY offered 18–22 weekly group sessions of 2–2.5 h. In the effectiveness trial phase, facilitators attended a 4 or 5 days manualised workshop and received supervision in the form of peer support meetings and monthly telephone consultations from accredited mentors. The majority of facilitators had a higher education level qualification in an education or health and social care discipline. Forty-seven parents (3.4%) enrolled in IY during the effectiveness trial phase, compared to 10 (0.27%) parents in the sustained implementation phase.

#### Triple P

The Triple P program (Sanders et al., 2000) was developed for parents of children aged 0–16 years old. It aims to increase parents’ skills and confidence in handling their child’s behavior though positive parenting. In the current study, providers of Triple P offered the Level 4 version which involves eight 2-h weekly sessions: four face-to-face small group sessions, three telephone sessions and one final group session. In the effectiveness trial phase, facilitators received a 3-day manualised training program with an accredited Triple P trainer. The majority of facilitators had a higher education level qualification in an education or health and social care discipline. The majority of parents attended Triple P: 1241 (89.28%) parents in the effectiveness trial phase, and 3442 (92.88%) parents in the sustained implementation phase.

#### STOP

STOP (Ministry of Parenting, 2015) was developed as an 11-week program for parents of children aged 11–16. It aims to help parents to communicate better with their children and to support their development, through discussions, role play and feedback to develop more effective parenting techniques. In the effectiveness trial phase, facilitators attended a 3-day workshop and received manualised training materials. In the effectiveness trial phase, 102 (7.34%) parents enrolled in STOP compared to 254 (6.85%) in the sustained implementation phase.

#### Program Fidelity

During the sustained implementation phase, in addition to existing trained staff, new facilitators received the same training as those engaged during the effectiveness trial phase. Both existing and newly trained facilitators received the same pattern of support and supervision as during the effectiveness trial stage. The monitoring of the implementation of programs by the facilitators was undertaken in the same manner during both phases according to each program’s specifications by senior LA staff, who were both trained and experienced in the program’s delivery. The criteria for completion of each program were the same in each phase, namely a minimum attendance of 75% of the group sessions, including the final session when the post-testing also occurred. This criterion was employed by the LA services during both the trial phase (Parent Early Intervention Programme) and during the sustained implementation phase. Facilitators completed a monitoring sheet after the final group session indicating those parents who had completed their course and also the reasons, if known, of parents who did not complete their course; these monitoring sheets were returned to the research team with the completed post-course questionnaires.

### Procedure

Procedure was the same during both phases. Parents completed a pre-test questionnaire booklet either at the start of the first session or at an introductory session before the parenting course commenced. Post-test data were collected in the last session of the program. All pre and post measures were distributed by the program facilitator and posted to the research team for analysis. Twelve months following the pre-test measures follow up questionnaires were posted to parents directly from the research center.

#### Analytic Strategy

Data involved repeated measurements, which were also nested within LAs. Therefore, we accounted for data non-independence using Generalized Estimating Equations (GEE; Liang and Zeger, 1986). With their focus on population average effects (Hubbard et al., 2010), GEEs provided a good match to the research question.

Missing data ranged from 26.56 to 44.62% at post-test and 82.21–85.97% at follow up. This high level of data loss is a common occurrence in community evaluations (McWey et al., 2015; Abrahamse et al., 2016). We examined the association between missing data, initial levels of child behavior problems and participant characteristics, and we found no association (analysis available on request). There was also no significant difference in the proportion of missing data between the phases, across all outcome measures (all *p* > 0.05, full analysis available on request). This therefore suggests that the mechanism of missing data was not systematically related either to participant characteristics or the intervention on offer (Schafer and Graham, 2002). We had no reason to reject that data were Missing Completely at Random (MCAR) and, as such, were appropriate for fitting into GEEs. To address missing data, a quasi-likelihood estimation was used (Liang and Zeger, 1986) that makes full use of information available. GEEs were fitted specifying an identity link and exchangeable working correlation matrix, with a robust estimator for the covariance matrix which yields accurate standard errors (Garson, 2013). Outcomes were standardized and continuous covariates were grand mean centered. GEEs controlled for data clustering at LA level and program level.

## Results

Generalized Estimating Equations were fitted for each outcome to examine whether the effect of study phase (effectiveness trial vs. sustained implementation), time (pre-test, post-test, follow up) or their interaction was significant, accounting for the effect of LA, parent program type, child age, child gender, child’s SEN status, parent gender, parent support need, ethnicity, and socio-economic deprivation. GEEs use a Wald chi-square test to test whether each predictor makes a significant overall contribution to the model, and then provide an estimated coefficient for each level of the predictor. Unadjusted descriptive statistics are shown in Table 2, while Tables 3, 4 present the GEE coefficients of each level of predictor variables. Below, we report the overall predictor effects.

**Table 2:** Means and standard deviations (in brackets) for all outcome measures.

		Effectiveness trial			Sustained implementation		
		Pre	Post	Follow up	Pre	Post	Follow up
**SDQ^1^** **(2–17 age group)**	*N*	1195	680	206	3097	1798	442
Conduct problems		4.04 (2.35)	2.88 (2.13)	2.99 (2.28)	4.07 (2.28)	2.88 (2.13)	2.83 (2.29)
Emotional symptoms		3.42 (2.54)	2.49 (2.35)	2.7 (2.46)	3.55 (2.61)	2.57 (2.4)	2.6 (2.60)
Hyperactivity		5.94 (2.59)	4.81 (2.55)	4.75 (2.56)	6.15 (2.62)	5.02 (2.56)	4.86 (2.82)
SDQ total difficulties		16.57 (6.99)	12.95 (6.90)	12.93 (7.49)	17.11 (7.05)	13.17 (7.01)	12.86 (7.82)
**Parenting Scale**	*N*	1324	755	235	2789	2053	449
Laxness		21.94 (6.77)	15.84 (6.31)	17.37 (6.34)	20.49 (7.97)	15.93 (6.42)	15.64 (6.16)
Over-reactivity		22.23 (6.95)	16.1 (6.46)	18.11 (6.21)	19.96 (7.87)	15.67 (6.09)	15.69 (6.16)
**WEMWBS^2^**	*N*	1358	752	232	3584	2071	503
		45.52 (10.63)	53.78 (9.20)	50.4 (11.03)	45.51 (10.83)	53.69 (9.23)	51.68 (10.50)


*^*1*^SDQ, Strengths and Difficulties Questionnaire.*

*^*2*^WEMWBS, Warwick and Edinburgh Mental Wellbeing Scale.*

**Table 3:** GEE y-standardized coefficients for child behavior problems^1^.

		Conduct disorder	Emotional symptoms	Hyperactivity	Total difficulties

		β *(SE)*	β *(SE)*	β *(SE)*	β *(SE)*
Time^∗^Phase interaction (ref: sustained implementation phase)	Pre	–0.06 (0.03)	0.07 (0.03)	–0.01 (0.03)	0.02 (0.03)
	Post test	–0.02 (0.04)	0.05 (0.04)	0.02 (0.04)	0.02 (0.04)
	Follow up	–0.01 (0.06)	0.12 (0.07)	–0.003 (0.06)	0.09 (0.06)
Child related factors	Child age	0.03 (0.003)^∗∗∗^	0.05 (0.004)^∗∗∗^	–0.02 (0.004)^∗∗∗^	0.03 (0.004)^∗∗∗^
	Female child	–0.14 (0.03)^∗∗∗^	0.08 (0.03)^∗∗^	–0.34 (0.03)^∗∗∗^	–0.18 (0.03)^∗∗∗^
	SEN	0.17 (0.04)^∗∗∗^	0.34 (0.05)^∗∗∗^	0.49 (0.04)^∗∗∗^	0.51 (0.04)^∗∗∗^
Parent related factors	Female parent	0.04 (0.04)	0.07 (0.04)	0.02 (0.04)	0.07 (0.04)
	Health need	0.13 (0.01)^∗∗∗^	0.15 (0.01)^∗∗∗^	0.10 (0.01)^∗∗∗^	0.16 (0.014)^∗∗∗^
	White British	0.28 (0.04)^∗∗∗^	0.02 (0.04)	0.24 (0.04)^∗∗∗^	0.17 (0.04)^∗∗∗^
	Socio-economic deprivation	0.07 (0.01)^∗∗∗^	0.04 (0.01)^∗∗^	0.05 (0.01)^∗∗∗^	0.06 (0.01)^∗∗∗^


*^*1*^Data controlled for parent program type and LA.*

*^∗^p < 0.05, ^∗∗^p < 0.01, ^∗∗∗^p < 0.001.*

**Table 4:** GEE y-standardized coefficients for parent outcomes^1^.

		Laxness	Over-reactivity	WEMWBS	

		β *(SE)*	β *(SE)*	β *(SE)*	
Time^∗^ Phase interaction (ref: sustained implementation phase)	Pre	–0.12 (0.04)^∗∗^	–0.27 (0.04)^∗∗∗^	0.12 (0.03)^∗∗∗^	
	Post test	0.07 (0.04)	–0.01 (0.04)	0.06 (0.04)	
	Follow up	–0.10 (0.06)	–0.23 (0.06)^∗∗∗^	0.19 (0.07)^∗∗^	
Child related factors	Child age	0.01 (0.004)	0.03 (0.004)^∗∗∗^	–0.004 (0.003)	
	Female child	0.01 (0.03)	–0.02 (0.03)	0.05 (0.03)^∗^	
	SEN	–0.02 (0.05)	–0.05 (0.04)	–0.08 (0.04)^∗^	
Parent related factors	Female parent	0.10 (0.04)^∗∗^	0.14 (0.04)^∗∗∗^	–0.19 (0.03)^∗∗∗^	
	Healthcare need	–0.01 (0.01)	0.04 (0.01)^∗∗^	–0.13 (0.01)^∗∗∗^	
	White British	–0.08 (0.05)	–0.06 (0.04)	–0.24 (0.04)^∗∗∗^	
	Socio-economic deprivation	0.09 (0.01)^∗∗∗^	–0.01 (0.01)	–0.05 (0.01)^∗∗∗^	


**^*1*^Data controlled for parent program type and LA.**

*^∗^p < 0.05, ^∗∗^p < 0.01, ^∗∗∗^p < 0.001*.

### Child Behavior Problems

Generalized Estimating Equations were fitted twice with SDQ data: once for children aged 4–17 years and once for children aged 2–17 years. As the results were very similar, we report findings from the 2–17 year-old group analysis. Results from the 4–17 years old group are available on request to the first author.

Overall, there was a significant time effect for each behavior problem: conduct problems (Wald = 849.73, *p* < 0.001), emotional symptoms (Wald = 441.66, *p* < 0.001), hyperactivity (Wald = 590.27, *p* < 0.001), total difficulties (Wald = 1033.87, *p* < 0.001). There was no significant effect of phase for conduct problems (Wald = 0.85, *p* = 0.358), hyperactivity (Wald = 0.01, *p* = 0.930), and total difficulties (Wald = 1.53, *p* = 0.217). Emotional symptoms were slightly higher in the sustained implementation phase (Wald = 5.09, *p* = 0.024).

There was also no significant interaction between time and phase for all subscales: conduct problems (Wald = 1.64, *p* = 0.441), emotional symptoms (Wald = 1.10, *p* = 0.576), hyperactivity (Wald = 0.73, *p* = 0.693), and total difficulties (Wald = 1.37, *p* = 0.503). These findings suggest that all child behavior problems decreased significantly from pre- to post-test, and pre- to follow up and that the decrease was similar across the two phases of this study.

Table 3 presents estimated coefficients for each level of the predictor variables; these can be interpreted as y-standardized regression coefficients. Child male gender (compared to female gender) was associated with significantly higher scores on conduct problems (β = -0.14, *p* < 0.001), hyperactivity (β = -0.34, *p* < 0.001) and total difficulties (β = -0.18, *p* < 0.001). Child female gender was associated with significantly higher scores on emotional symptoms than male children (β = 0.08, *p* = 0.003). Child age was positively associated with conduct problems (β = 0.03, *p* < 0.001), emotional symptoms (β = 0.05, *p* < 0.001) and total difficulties (β = 0.03, *p* < 0.001), but negatively with hyperactivity (β = -0.02, *p* < 0.001). Having SEN was associated with higher scores on conduct problems (β = 0.17, *p* < 0.001), emotional symptoms (β = 0.34, *p* < 0.001), hyperactivity (β = 0.49, *p* < 0.001), and total difficulties (β = 0.51, *p* < 0.001).

There were no significant associations of parent gender with any child behavior problem (Table 3), but parents with higher support needs rated their children higher on conduct problems (β = 0.13, *p* < 0.001), emotional symptoms (β = 0.15, *p* < 0.001), hyperactivity (β = 0.10, *p* < 0.001) and total difficulties (β = 0.16, *p* < 0.001). Socio-economic deprivation was associated with higher levels of conduct problems (β = 0.07, *p* < 0.001), emotional symptoms (β = 0.04, *p* = 0.001), hyperactivity (β = 0.05, *p* < 0.001), and total difficulties (β = 0.06, *p* < 0.001). Compared to all other ethnic groups, White British ethnicity was associated with higher scores on conduct problems (*β* = 0.28, *p* < 0.001), hyperactivity (β = 0.24, *p* < 0.001) and total difficulties (β = 0.17, *p* < 0.001), but was unrelated to emotional symptoms (β = 0.02, *p* = 0.718).

### Parenting

**FIGURE 2:**
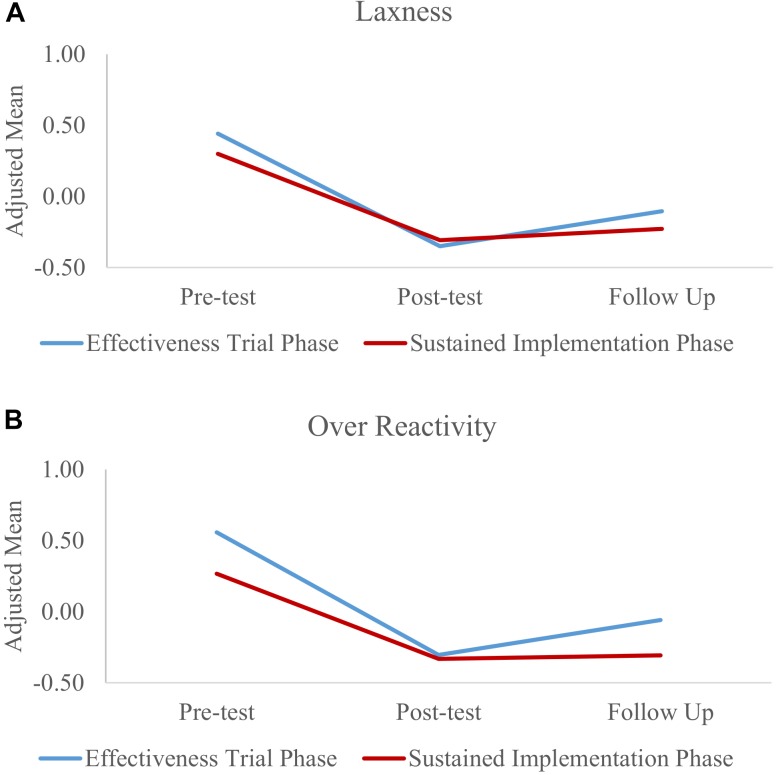
Standardized adjusted means for Parenting Style scores **(A)** laxness, **(B)** over reactivity.

The interactions between time and phase were significant for laxness (Wald = 23.22, *p* < 0.001) and over-reactivity (Wald = 42.29, *p* < 0.001). To interpret the significant interactions, we focus on each time^∗^phase coefficient. At pre-test, scores of both parenting styles scores were significantly lower in the sustained implementation phase compared to the effectiveness trial phase (laxness: β = -0.12, *p* = 0.001; over-reactivity: β = -0.27, *p* < 0.001). At post-test, differences between the two phases were no longer significant: laxness (β = 0.04, *p* = 0.254; over-reactivity: β = -0.03, *p* = 0.462). Follow up scores indicated better maintenance of gains in the sustained implementation phase for over-reactivity (β = -0.23, *p* < 0.001), but not laxness (β = -0.10, *p* = 0.086). Figure 2 presents adjusted laxness and over-reactivity scores over time for each phase that demonstrate the significant interaction described above.

Laxness scores were not related to child gender (β = 0.01, *p* = 0.595), child age (β = 0.01, *p* = 0.209) or SEN status (β = -0.02, *p* = 0.737). Over-reactivity scores were higher for older children (β = 0.03, *p* < 0.001), but there was no association with child gender (β = -0.02, *p* = 0.501) or the child’s SEN status (β = -0.05, *p* = 0.231). Mothers had higher scores on laxness (β = 0.10, *p* = 0.004) and over-reactivity (β = 0.14, *p* < 0.001) than fathers. Parent support need was not significantly related to laxness (β = -0.01, *p* = 0.44), but there was an association with over-reactivity (β = 0.04, *p* = 0.007). Ethnicity was not significantly associated with either laxness or over-reactivity (laxness: β = -0.08, *p* = 0.094, over-reactivity: β = -0.06, *p* = 0.211). Socio-economic deprivation was significantly associated with higher laxness (β = 0.09, *p* < 0.001) but not associated with over-reactivity (β = -0.01, *p* = 0.430).

### Parent Mental Well-Being

The main effect of time on WEMWBS scores was significant (Wald = 1649.99, *p* < 0.001), indicating an increase in mental well-being from pre-test to post-test and from pre-test to follow up. Mental well-being scores were significantly higher in the sustained implementation phase compared to the effectiveness trial phase (Wald = 12.96, *p* < 0.001) but the interaction between time and phase was not significant (Wald = 4.93, *p* = 0.085), suggesting that, despite group differences, there was no differential gain between the two phases: they both experienced a significant improvement over time.

Generalized Estimating Equations coefficients are presented in Table 4. Having a female child who was causing concern was associated with higher parent mental well-being scores (β = 0.05, *p* = 0.046). Child’s SEN status was associated with lower mental well-being scores (β = -0.08, *p* = 0.047). Parent mental well-being was not related to child age (β = -0.004, *p* = 0.235). Mental well-being scores were higher for male parents (β = -0.19, *p* < 0.001) and for parents with lower levels of support need (β = -0.13, *p* < 0.001). Parent mental well-being was lower for those with a higher level of socio-economic deprivation (β = -0.05, *p* < 0.001) and lower for those of White British ethnicity (β = -0.24, *p* < 0.001).

## Discussion

The current study examined whether evidence-based parenting programs (EBPPs) remain effective when delivered entirely by service providers as part of regular service delivery during the sustained implementation phase. Findings indicated that service-led sustained implementation was associated with significant improvements in child behavior problems, similar to the researcher-led effectiveness trial; there were significant improvements in parenting style, which were larger than the effectiveness trial at 12 months follow up; and significant improvements in parental mental well-being similar to the effectiveness trial.

Present findings come from a large English sample of parents and support findings from an implementation trial in the United States (Price et al., 2012), where researchers compared service-led implementation of a parenting group for foster carers with the comparison group of a previous effectiveness trial, and found the implementation phase resulted in significant benefits in child behavior problems. Present findings provide a rigorous demonstration of successful maintenance of the effectiveness of EBPPs delivered during the sustained implementation phase, relative to a group of people who also received EBPPs during the effectiveness trial.

Significant gains immediately after the program were maintained at 12 months follow up, similarly to the initial improvement and maintenance demonstrated in the effectiveness trial. Indeed, longer-term gains in parenting style (laxness and over-reactivity) were larger for parents in service-led implementation. This could be because parents who took up parenting programs during service implementation presented lower levels of initial difficulties. The present findings add to the limited evidence about the longer-term effectiveness of parenting programs, whether in community provision or research evaluations (c.f., Lundahl et al., 2006).

In the current study, we did not measure the factors that change between a researcher-led trial and service-led evaluation, but we know that a large number of conditions may change as we move from researcher-led evaluations to service-led delivery (Durlak and DuPre, 2008). Our aim was to examine whether effectiveness would be maintained, despite the changes in the implementation environment and circumstances. Our findings indicated that effectiveness can be maintained, though without identifying factors that are important for the maintenance of effectiveness. As our understanding of the implementation continuum improves (Spoth et al., 2013; Gottfredson et al., 2015), it is obvious that we have a patchy understanding of all conditions related to successful sustained implementation (Proctor, 2009; Wiltsey Stirman et al., 2012). The issue of effectiveness as an indicator of achieving population level benefits should be a core outcome of the sustained implementation agenda, beyond the knowledge that each service provider requires to monitor their quality. The current study demonstrated the maintenance of implementation effectiveness in a large English sample. Future research should examine further the service-level factors that may be related to successful effectiveness during sustained implementation.

The present data also suggested some interesting differences in terms of the parents who received programs across the two phases. While both phases operated a targeted provision model, parents who signed up during the sustained implementation phase experienced lower levels of parenting difficulties and better mental well-being before the programs started, compared to parents in the effectiveness trial. Though there were more White British parents and less income poverty (fewer children eligible for free school meals) in the sustained implementation phase, there were overall similar levels of socio-economic deprivation, child characteristics and child behavior problems (see Table 1). Therefore initial differences might not be entirely accounted for by socio-demographic differences. These differences may indicate differences in the way services recruit or refer parents over time, i.e., a broadening of referral criteria to a larger population during sustained implementation.

One of the core limitations of the present study is the self-selected sample of areas from which sustained implementation data were pooled. These were LAs where service providers decided to continue delivering and monitoring parenting programs following their involvement in the researcher-led effectiveness trial, and they may differ from other LAs who did not choose to continue independent evaluation of the effectiveness of the implementation of their EBPPs after the effectiveness trial, following the end of funding of the Parent Early Intervention Programme by the DfE. In particular, these LAs were committed to maintaining staff training, support and program fidelity; as well as monitoring of effectiveness by an independent research team, so providing accountability. Future research needs to investigate further the in-depth factors that facilitate or hinder sustained service implementation following a successful take-up.

A further limitation of the present study concerns the generalizability of the findings, due to the high level of data attrition. While common in community-based research (McWey et al., 2015; Abrahamse et al., 2016), this limits our conclusions to those parents for whom evaluation data were available, rather than all parents who enrolled for the programs. There was limited information with regards to the specific reasons for this level of data attrition for the LAs featured in the current study, although our analysis did not point to a single reason (mechanism) for missing data: reasons included parents’ practical difficulties related to family factors, (e.g., illness, husband’s change of work pattern). However, given the high level of missing data, it is important that future research focuses on understanding patterns of attrition in users of community services to enhance the likelihood of continued engagement with programs and evaluation.

## Conclusion

Research demonstrating the sustainability of EBPPs’ effectiveness is of key importance in determining their funding-worth (August et al., 2006; Wiltsey Stirman et al., 2012). However, the focus of much sustainability research has been on optimal implementation environments, rather than the maintenance of outcomes and the demonstration of benefit across the population as a whole. The current study demonstrated that effectiveness can be maintained when services lead on provision of EBPPs. Present findings indicated that improvements in child behavior problems and parental mental well-being were significantly maintained during sustained implementation, whereas improvements in parenting laxness and over-reactivity were significant in the short-term but better maintained in the longer term under sustained implementation. Given the high costs of late intervention (Chowdry and Fitzsimons, 2016), the present findings make a strong case for the integration of EBPPs in public health approaches to reducing child behavior problems and parent well-being.

## Ethics Statement

This study was carried out in accordance with the recommendations of the Humanities and Social Sciences Research Ethics Committee of the University of Warwick. The protocol was approved by the Humanities and Social Sciences Research Ethics Committee of the University of Warwick [Ref: Eth. App. 12/06-07, (Lindsay et al., 2011b); Ref: Eth. App. 45/07-08, (Lindsay and Strand, 2013); Ref: Eth. App. 93/15-16, for the current study]. All subjects gave written informed consent in accordance with the Declaration of Helsinki.

## Author Contributions

VT, GG, and GL substantial contributions to the conception or design of the work, or the acquisition, analysis, or interpretation of data for the work, drafting the work or revising it critically for important intellectual content, final approval of the version to be published, agreement to be accountable for all aspects of the work in ensuring that questions related to the accuracy or integrity of any part of the work are appropriately investigated and resolved.

## Conflict of Interest Statement

The authors declare that the research was conducted in the absence of any commercial or financial relationships that could be construed as a potential conflict of interest.

## Footnotes

1 www.sdqinfo.com/norms/UK3yearNorm.html
